# A new approach to epigenome-wide discovery of non-invasive methylation biomarkers for colorectal cancer screening in circulating cell-free DNA using pooled samples

**DOI:** 10.1186/s13148-018-0487-y

**Published:** 2018-04-16

**Authors:** María Gallardo-Gómez, Sebastian Moran, María Páez de la Cadena, Vicenta Soledad Martínez-Zorzano, Francisco Javier Rodríguez-Berrocal, Mar Rodríguez-Girondo, Manel Esteller, Joaquín Cubiella, Luis Bujanda, Antoni Castells, Francesc Balaguer, Rodrigo Jover, Loretta De Chiara

**Affiliations:** 1 Department of Biochemistry, Genetics and Immunology, Centro Singular de Investigación de Galicia (CINBIO), University of Vigo, Campus As Lagoas-Marcosende s/n, 36310 Vigo, Spain; 2 Cancer Epigenetics and Biology Program (PEBC), Bellvitge Biomedical Research Institute (IDIBELL), Barcelona, Spain; 3 Department of Medical Statistics and Bioinformatics, Leiden University Medical Centre, Leiden, The Netherlands; 4 SiDOR Research Group and Centro de Investigaciones Biomédicas (CINBIO), Faculty of Economics and Business Administration, University of Vigo, Vigo, Spain; 5 Department of Gastroenterology, Complexo Hospitalario Universitario de Ourense, Instituto de Investigación Biomédica Galicia Sur, Centro de Investigación Biomédica en Red de Enfermedades Hepáticas y Digestivas (CIBERehd), Ourense, Spain; 6 Department of Gastroenterology, Instituto Biodonostia, Centro de Investigación Biomédica en Red de Enfermedades Hepáticas y Digestivas (CIBERehd), Universidad del País Vasco (UPV/EHU), San Sebastián, Spain; 7 Gastroenterology Department, Hospital Clínic, IDIBAPS, CIBERehd, University of Barcelona, Barcelona, Spain; 8 Department of Gastroenterology, Hospital General Universitario de Alicante, Alicante, Spain

**Keywords:** Advanced adenomas, Circulating cell-free DNA, Colorectal cancer, DNA methylation, MethylationEPIC, Non-invasive diagnostic biomarkers, Pooled samples, Serum

## Abstract

**Background:**

Colorectal cancer is the fourth cause of cancer-related deaths worldwide, though detection at early stages associates with good prognosis. Thus, there is a clear demand for novel non-invasive tests for the early detection of colorectal cancer and premalignant advanced adenomas, to be used in population-wide screening programs. Aberrant DNA methylation detected in liquid biopsies, such as serum circulating cell-free DNA (cfDNA), is a promising source of non-invasive biomarkers. This study aimed to assess the feasibility of using cfDNA pooled samples to identify potential serum methylation biomarkers for the detection of advanced colorectal neoplasia (colorectal cancer or advanced adenomas) using microarray-based technology.

**Results:**

cfDNA was extracted from serum samples from 20 individuals with no colorectal findings, 20 patients with advanced adenomas, and 20 patients with colorectal cancer (stages I and II). Two pooled samples were prepared for each pathological group using equal amounts of cfDNA from 10 individuals, sex-, age-, and recruitment hospital-matched. We measured the methylation levels of 866,836 CpG positions across the genome using the MethylationEPIC array. Pooled serum cfDNA methylation data meets the quality requirements. The proportion of detected CpG in all pools (> 99% with detection *p* value < 0.01) exceeded Illumina Infinium methylation data quality metrics of the number of sites detected. The differential methylation analysis revealed 1384 CpG sites (5% false discovery rate) with at least 10% difference in the methylation level between no colorectal findings controls and advanced neoplasia, the majority of which were hypomethylated. Unsupervised clustering showed that cfDNA methylation patterns can distinguish advanced neoplasia from healthy controls, as well as separate tumor tissue from healthy mucosa in an independent dataset. We also observed that advanced adenomas and stage I/II colorectal cancer methylation profiles, grouped as advanced neoplasia, are largely homogenous and clustered close together.

**Conclusions:**

This preliminary study shows the viability of microarray-based methylation biomarker discovery using pooled serum cfDNA samples as an alternative approach to tissue specimens. Our strategy sets an open door for deciphering new non-invasive biomarkers not only for colorectal cancer detection, but also for other types of cancers.

**Electronic supplementary material:**

The online version of this article (10.1186/s13148-018-0487-y) contains supplementary material, which is available to authorized users.

## Background

Colorectal cancer (CRC) is the fourth leading cause of cancer-related deaths worldwide, accounting for over 1.4 million new cases in 2012 [1, 2]. While diagnosis at early stages associates with good prognosis and reduced mortality rates, the detection and removal of premalignant advanced adenomas (AA) results in the reduction of CRC incidence [3]. Since neoplastic transformation can last decades, there is a broad time window for implementing screening strategies for the detection of advanced neoplasia (AN: CRC or AA) [3, 4].

Approaches for CRC screening can be divided into two groups. Invasive procedures like colonoscopy allow the examination of the entire colon and the removal of lesions (polypectomy); however, limitations of this strategy include considerably low participation rates and high cost [5]. On the other hand, non-invasive methods like fecal immunological test (FIT) have the advantage of increased acceptance and adequate specificity, though sensitivity for colorectal tumors, especially of proximal location, and AA is moderate to low [6, 7]. Blood-based markers are capable of improving CRC screening adherence, and a large number of candidates have been reported for CRC diagnosis, reviewed in [8]. Currently, the most promising is the *SEPT9* methylation assay, though its performance for the detection of early-stage tumors and AA needs to be improved [9]. Therefore, there is an imperative need of finding new non-invasive biomarkers for CRC screening.

Nowadays, it is well-established that not only genetic alterations but also epigenetic modifications are involved in CRC development and progression [10]. The abnormal methylation occurring during colorectal neoplasia is characterized by promoter hypermethylation and transcriptional silencing of tumor suppressor or DNA repair genes [11, 12], coexisting with a global loss of methylation that leads to chromosomal and microsatellite instability and oncogene activation [11, 13]. Both promoter hypermethylation and global hypomethylation are hallmarks of early stages of colorectal carcinogenesis [10, 14].

Several methodologies are suitable for genome-wide methylation biomarker discovery, including whole and reduced genome bisulfite sequencing and array-based genotyping technology [15, 16]. These epigenome-wide measurements allow a more successful identification of methylation alterations related to complex diseases compared to target studies. The main drawback is the large sample size needed, which increases project costs. DNA sample pooling strategies represent an affordable approach for biomarker discovery, resulting in reduced costs and increased amount of input DNA when small amounts are available. Additionally, it has been reported that pooled samples provide similar results to individual samples in both genome-wide [17, 18] and epigenome-wide [19] association studies.

During the last years, it has been demonstrated that circulating cell-free DNA (cfDNA) present in liquid biopsies reflects methylation changes originated in tumor cells [20–22]. Given the stability of DNA methylation in body fluids [23–25], the discovery of cfDNA methylation markers using serum samples seems a very attractive alternative to direct the search of non-invasive biomarkers.

Taking advantage of this fact, we hypothesized that an array-based epigenome-wide analysis using serum cfDNA as input could be a novel and affordable approach for the discovery of a methylation marker panel with greater diagnostic value, compared to other indirect strategies using tumor tissue and mucosa as input DNA. Therefore, in the present study, we aim to assess the feasibility of hybridizing pooled serum cfDNA to the MethylationEPIC array to detect differentially methylated patterns between patients with advanced neoplasia and individuals with no colorectal findings.

## Methods

### Study population and serum samples

Individuals were recruited from the following Spanish Hospitals: Hospital Donostia (San Sebastián), Complexo Hospitalario Universitario de Ourense (Ourense), Hospital Clínic de Barcelona (Barcelona), and Hospital General Universitario de Alicante (Alicante). Patients’ characteristics are described elsewhere [26]. We carried out a stratified random sampling using colorectal finding and gender as stratifying variables. Moreover, age was restricted to 50–75 years and strata were matched by recruitment hospital and age. We selected from this multicenter cohort 20 individuals with no colorectal findings (NCF), 20 individuals with AA (adenomas ≥ 10 mm, with villous component or high-grade dysplasia), and 20 CRC cases (7 stage I and 13 stage II, according to the AJCC staging system [27]). Individuals were classified according to the most advanced lesion after colonoscopy. Lesions were considered “proximal” when located only proximal to the splenic flexure of the colon and “distal” when lesions were found only in the distal colon or in both distal and proximal colons. Advanced neoplasia (AN) was defined as AA or CRC.

Blood samples were obtained the same day of the colonoscopy, immediately prior to the procedure. Blood samples were coagulated and subsequently centrifuged according to the manufacturer’s instruction for serum collection. Serum samples were stored at − 20 °C until used.

### DNA extraction and sample pooling

We extracted cfDNA from 0.5–1.5 mL of serum using a phenol-chloroform protocol as described by Clemens et al. [28], with minor modifications, and resuspended in 20 μL sterile water. DNA concentration was determined for each individual sample using the Qubit dsDNA HS Assay Kit (Thermo Fisher Scientific, MA, USA), a fluorimetric assay specific for double-stranded DNA that gives an accurate measurement of DNA concentration. All cfDNA samples were stored at − 20 °C.

Two independent pooled samples were constructed for each pathological group (NCF, AA, and CRC) using equal amounts of cfDNA from 10 individuals per pool. The factors considered to match between pools were gender, age, and recruitment hospital. Table 1 shows epidemiologic and clinical data of each individual included in pool A and B (NCF), pool C and D (AA), and pool E and F (CRC).

**Table 1:** Epidemiologic and clinical characteristics of the individuals included in the pools

Pool^a^	Gender^b^	Age		Gender^b^	Age
Pool A-NCF	F	72	Pool B-NCF	F	63
	F	68		F	62
	F	61		F	59
	F	54		F	54
	F	54		F	54
	M	67		M	71
	M	67		M	68
	M	65		M	66
	M	62		M	60
	M	54		M	53
	Gender^b^	Age	Lesion description^c^	Lesion location^d^	
Pool C-AA	F	72	10 mm, T, LGD	Distal	
	F	68	25 mm, V, LGD	Distal	
	F	65	10 mm, TV, LGD	Distal	
	F	63	12 mm, T, LGD	Proximal	
	F	54	10 mm, T, LGD	Distal	
	M	71	10 mm, T, LGD	Distal	
	M	66	10 mm, TV, LGD	Distal	
	M	65	20 mm, T, LGD	Distal	
	M	61	15 mm, T, LGD	Proximal	
	M	58	3 mm, TV, LGD	Proximal	
Pool D-AA	F	70	30 mm, T, LGD	Proximal	
	F	67	20 mm, TV, LGD	Distal	
	F	65	30 mm, TV, LGD	Proximal	
	F	61	10 mm, TV, LGD	Distal	
	F	59	10 mm, TV, LGD	Distal	
	M	71	8 mm, TV, LGD	Distal	
	M	71	10 mm, V, LGD	Proximal	
	M	64	20 mm, V, LGD	Distal	
	M	64	20 mm, T, LGD	Proximal	
	M	54	5 mm, TV, LGD	Distal	
Pool E-CRC	F	70	T3N0, WD	Distal	
	F	67	T3N0, MD	Distal	
	F	65	T3N0, MD	Distal	
	F	59	T4N0, MD	Distal	
	F	59	T3N0, WD	Distal	
	M	72	T2N0, MD	Distal	
	M	66	T2N0, NA	Distal	
	M	62	T3N0, MD	Distal	
	M	60	T1N0M0, WD	Distal	
	M	51	T3N0, MD	Proximal	
Pool F-CRC	F	72	T3N0, WD	Distal	
	F	65	T3N0, WD	Distal	
	F	61	T2N0, MD	Distal	
	F	60	T3N0, MD	Proximal	
	F	55	T3N0, NA	Distal	
	M	67	T2N0M0, WD	Proximal	
	M	63	T2N0, MD	Distal	
	M	61	T3N0, MD	Distal	
	M	59	T3N0, WD	Distal	
	M	57	T2N0, MD	Distal	

^a^Identification of the pool (*NCF* no colorectal findings, *AA* advanced adenoma, *CRC* colorectal cancer)

^b^Gender (*F* female, *M* male)

^c^Lesion description for AA cases include size of adenoma (mm), histology (*T* tubular, *TV* tubulo-villous, *V* villous) and dysplasia (*LGD* low grade of dysplasia), lesion description for CRC cases include TNM classification and tumor differentiation grade (*WD* well-differentiated, *MD* moderately differentiated, *NA* not available)

^d^Lesion location refers to distal or proximal colon

Since the preparation of pooled samples is a critical step that requires high accuracy, cfDNA from each individual included in a pool was thawed, tempered, and re-quantified using the Qubit assay. As reported by previous DNA pooling protocols [18, 29], in order to avoid inaccuracies derived from pipetting small volumes, we decided to dilute by a factor of two samples with more than 10 ng/μL of DNA. Diluted DNA was measured again.

Once the actual concentration of all the individual samples of a pool was available, we determined the sample containing the limiting ng of cfDNA (based on measured concentration and volume). Based on this limiting nanogram, we calculated for each of the nine remaining samples the volume containing the same nanogram of cfDNA as the limiting sample. Finally, the pool was constructed by incorporating into the tube the corresponding volume of each of the 10 individual samples of the pool. The cfDNA mixture was allowed to stand for 1 h, and then the DNA concentration was quantified with the Qubit Assay to ensure that the final DNA concentration of the pool was as expected according to the theoretical calculation:

$$ \frac{\left(\mathrm{limiting}\ \mathrm{ng}\right)\cdotp n}{\left(\mathrm{total}\ \mathrm{volume}\ \mathrm{of}\ \mathrm{the}\ \mathrm{pool}\right)} $$

where *n* is the number of individuals included in each pool (10). Pools were considered valid for the Infinium Methylation Assay protocol when the difference between expected and measured concentration (Qubit) was less than 5%. A graphical description of the pooling protocol is presented [see Additional file 1]. This protocol was followed for each of the pools included in the study. The six pooled cfDNA samples were stored at − 20 °C and were submitted to the Cancer Epigenetics and Biology Program (PEBC) facilities at the Bellvitge Biomedical Research Institute for processing.

### Epigenome-wide methylation measurements

DNA methylation was analyzed with the Infinium MethylationEPIC BeadChip microarray (EPIC; Illumina Inc., CA, USA), that quantitatively measures the methylation levels of more than 850,000 CpG sites across the genome [30], located in promoter regions and gene bodies, and also in intergenic enhancer regions identified by the ENCODE [31, 32] and FANTOM5 [33] projects. Pooled samples were bisulfite treated in the same batch, and MethylationEPIC arrays were hybridized according to manufacturer’s instructions.

### Data preprocessing and differential methylation analysis

Data quality control was assessed with the GenomeStudio V2011.1, based on the internal control probes present on the array. The preprocessing, normalization, and correction steps were conducted using the R environment (versions 3.3.3 and 3.4.0) with Bioconductor packages. The pipeline was a sequence of R functions adapted from the minfi [34] and ChAMP [35] Bioconductor packages. Our dataset was normalized using the Functional Normalization implemented in the minfi package. This algorithm does not rely on any biological assumption and therefore is suitable for cases where global changes in the methylation levels are expected, such as in cancer-normal comparisons [36].

Detection *p* values were computed with the minfi package, and mean detection *p* values were examined across all samples in order to identify any failed sample. Probes with a detection *p* value > 0.01 in at least one sample were discarded. We filtered out probes containing a single nucleotide polymorphism (SNP) at the CpG interrogation site and at the single nucleotide extension for any minor allele frequency (MAF), and probes containing a SNP at the probe body for a MAF >5 %, because differential methylation levels can be confounded with actual polymorphisms in the DNA sequence. According to the list provided by Pidsley et al. [37], cross-reactive probes were removed. Probes targeting X and Y chromosomes were also discarded.

In accordance with Du et al. [38], methylation levels were expressed as beta and *M* values. Beta values were used for visualization and intuitive interpretation of the results, and *M* values were used for the differential methylation analysis.

Prior to differential methylation analysis, data was checked for batch effects across all array runs using the combat method implemented in the ChAMP package. Differentially methylated positions (DMP) between NCF and AN (AA or CRC) were detected with the dmpFinder function from the minfi package, which uses an F-test for categorical phenotype comparisons at a probe level. *p* values for each probe were corrected for multiple testing using the Benjamini-Hochberg procedure, with a false discovery rate (FDR) of 5% to determine significant DMPs.

### In silico evaluation of differential methylation

We applied unsupervised clustering approaches to evaluate the differentially methylated patterns between AN and NCF pools in an independent dataset. The publicly available dataset GSE48684 that includes the methylation data of 64 colorectal tumor biopsies (adenocarcinomas) and 41 healthy mucosa biopsies, measured with the Infinium HumanMethylation450 BeadChip array (450K) [14], was used as a test cohort. This independent evaluation was limited to the probes shared by 450K and EPIC arrays due to the absence of colorectal tumor and mucosa EPIC public datasets.

## Results and discussion

### DNA pooling methodology

To our knowledge, this is the first pooling-based study that analyzes the methylation patterns in cfDNA, aiming to assess the feasibility of liquid biopsy methylation biomarker discovery using microarray technology in a more affordable manner compared to individual samples.

DNA sample pooling has been reported as an efficient tool for genome-wide and high-throughput association studies [17, 18]. More recently, its potential utility was highlighted in microarray-based epigenome-wide association studies (EWAS), as Gallego-Fabrega et al. reported high correlation of the methylation levels between pools and individual DNA samples using the Infinium HumanMethylation450 BeadChip [19]. Taking into account the limitation that only mean methylation levels can be obtained from pooled samples, pooling strategy is an accurate and affordable alternative that can significantly reduce costs in large EWAS. DNA pooling is also an efficient alternative when small amounts of DNA are available and when working with precious samples.

For sample pooling, accurate construction is critical, and each DNA sample must be equally represented in the pool. To guarantee the most precise pool construction, we first tested two different pooling strategies: diluting all samples to a common concentration and then mixing equal volumes in a tube as in previous works [19, 29] or directly adding the same nanogram of DNA (calculated corresponding volume from each sample) into the tube. Once test pools were constructed and DNA concentration measured, we checked for discrepancies between the actual and the expected concentration. Variations inferior to 5% of the expected pooled DNA concentration were found when using the second protocol; therefore, sample pooling was performed as described [see Additional file 1].

We prepared two pooled samples for each pathological group (two pools of individuals with NCF, two pools of AA patients, and two pools of CRC patients stages I and II). We included 10 individuals per pool to ensure an acceptable amount of DNA input for the microarray analyses and also to reduce population stratification and the presence of unobserved confounding variables. The categories considered to match between pools were gender, age (median 63.5, range 51–72 years), and recruitment hospital. The age range was selected based on the USPSTF guideline recommendation for CRC screening, targeting individuals from 50 to 75 years [39]. No statistically significant difference was found in the mean age between pools (ANOVA, *p* < 0.05). The final cfDNA concentration of the six pooled samples ranged from 135 to 250 ng.

### Quality control of methylation data

The methylation levels of 866,836 CpG positions across the genome in the six pooled samples were measured using the MethylationEPIC BeadChip. The quality control based on the internal control probes present on the array, which include bisulfite conversion efficiency, hybridization, extension, and staining, among others, indicates that pooled serum cfDNA methylation data meets the quality requirements. The QC report is presented [see Additional file 2] and shows that the signals observed are much higher than the background signal, coinciding with what is expected for high-quality DNA. In relation to CpG detection, all the pools showed more than 99% of CpG detected correctly (only 2811 probes presented a detection *p* value > 0.01 at least in one sample, and were discarded). The number of probes detected in each pool was 866,497; 866,021; 865,865; 865,501; 865,778; and 866,463 for pools A, B, C, D, E, and F, respectively. These results are indicative of a uniform amplification and hybridization in all the pooled samples. The proportion of CpG detection observed in our samples exceeded Illumina Infinium methylation data quality metrics of the number of sites detected (> 96% for genomic DNA and > 90% for FFPE samples). A probable explanation could be the pooling design, as measuring methylation of 10 individuals in the same assay would increase the representation of each CpG in the input DNA. Therefore, no samples were discarded due to QC issues.

**Fig. 1:**
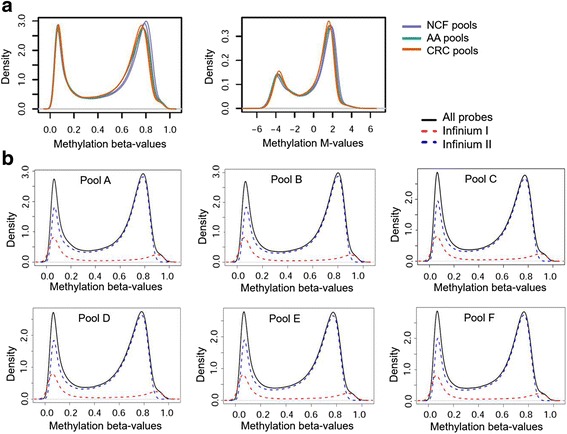
Density distributions of methylation data. **a** Density distribution of the raw methylation beta and *M* values across the 866,836 CpG sites measured in the six pooled serum cfDNA pooled samples. **b** Density distribution of the beta values by probe type for all the interrogated CpG sites in pools A–F

The distribution of methylation levels in pooled samples presented the expected bimodal distribution for both beta and *M* values, with the two peaks indicating fully methylated and unmethylated states characteristic of DNA methylation data (Fig. 1a). Then, we evaluated the distribution of beta values by type I and II probes separately. As observed in Fig. 1b, all the pools showed the distribution of type II probes shifted in relation to type I, as previously reported in the 450K and EPIC data [37, 40].

### Differential methylation

**Fig. 2:**
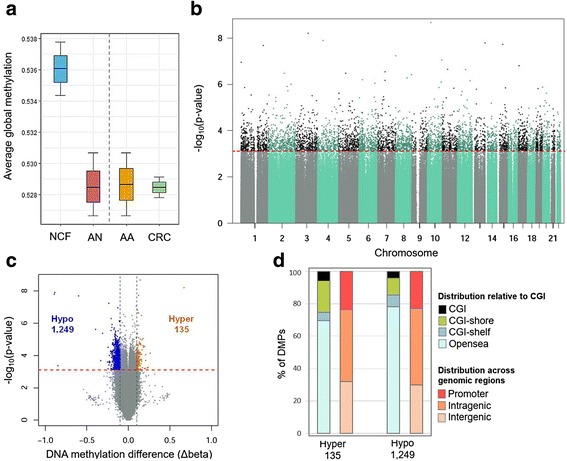
Identification of differential methylation. **a** Boxplot of global cfDNA methylation in NCF, AN, AA, and CRC pools. Global methylation is expressed as the average methylation rate for each pooled sample. The box plot represents the median (line across the box), interquartile range, and maximum and minimum values (whiskers). **b** Manhattan plot showing −log_10_(*p* value) resulting from the differential methylation analysis for all the CpGs considered (703,653). The *p* values are sorted by chromosome coordinates. Significant DMPs between AN and NCF pooled samples with a FDR < 5% (5808) appear highlighted in darker color, above the red dashed line. **c** Volcano plot of differential methylation −log_10_(*p* value) versus differences in methylation levels (Δbeta: obtained by subtracting the DNA methylation levels (beta values) of NCF from AN). Significant DMPs appear above the red dashed line (FDR 5%). Significant DMPs with a difference in the methylation levels greater than 10% (1384) are highlighted in color (135 hypermethylated DMPs in AN, orange dots: Δbeta > 0.1 and FDR < 5%; 1249 hypomethylated DMPs in AN, blue dots: Δbeta < − 0.1 and FDR < 5%). **d** Relative distribution of the 1385 DMPs with absolute Δbeta > 0.1 in relation to CpG islands (CGI) and across different genomic regions. The EPIC array categorizes probes following a functional classification into three major groups: promoter regions (5′UTR, TSS200, TSS1500, and first exons), intragenic regions (gene body and 3′UTR), and intergenic regions. TSS200, TSS1500: 200 and 1500 bp upstream the transcription start site, respectively. CGI-shore: sequences 2 kb flanking the CGI, CGI-shelf: sequences 2 kb flanking shore regions, opensea: sequences located outside these regions [30]

Once the technical quality of the six pooled sample data is verified, we performed a differential methylation analysis. In order to detect differentially methylated positions (DMPs), we compared the two NCF pools (colonoscopically confirmed controls) with the two AA pools together with the two CRC pools (considered as AN). The differential methylation analysis was performed on the 703,653 probes left after the filtering step (see the “Methods” section). We first assessed the global methylation in the NCF and AN groups, and in the AA and CRC groups. As shown in Fig. 2a, a lower content of global methylation was observed in AN, AA, and CRC compared to NCF. In addition, we found there is no difference in terms of global methylation between AA and CRC cfDNA pooled samples.

Since the purpose of screening programs include the detection of early stage CRC together with the identification and removal of premalignant AA [4], we grouped AA and CRC in the single group AN for the analyses. We found a total of 5808 significant DMPs between the NCF and AN groups, identified with a FDR of 5% (Fig. 2b). Of these, 1384 presented at least 10% difference in the methylation level between NCF and AN (|Δbeta| > 0.1): 135 (9.75%) were found hypermethylated in AN, while 1249 (90.25%) appeared hypomethylated (Fig. 2c). The distribution of the DMPs identified according to their location relative to CpG islands (CGI) and promoter regions is represented in Fig. 2d. The majority of the differentially hypomethylated CpGs in AN are located in opensea (78.08%), outside gene promoters, within regions with no enrichment in CpG content followed by CGI-shore (10.23%), CGI-shelf (7.50%), and CGI (4.19%).

DNA hypomethylation was the first aberrant methylation alteration described in several human cancers (reviewed in [41]). This global loss of genome-wide methylation was also described long time ago in both CRC and colorectal adenomas [42], indicating that global hypomethylation is characteristic of early stages of colorectal carcinogenesis [10, 14, 43]. Large hypomethylated blocks were also identified by Timp and colleagues (2014) using the 450K array. Among the six different tumor types analyzed, colon cancer tissue showed the highest proportions of hypomethylation in opensea, CGI-shelf, CGI-shore, and CGI [44]. In our work, using pooled serum samples, we found that more than 90% of the DMPs appeared hypomethylated in AN, agreeing with these previous reports, and suggesting that perhaps efforts should be centered on hypomethylated candidates to accomplish a greater discrimination capacity.

**Fig. 3:**
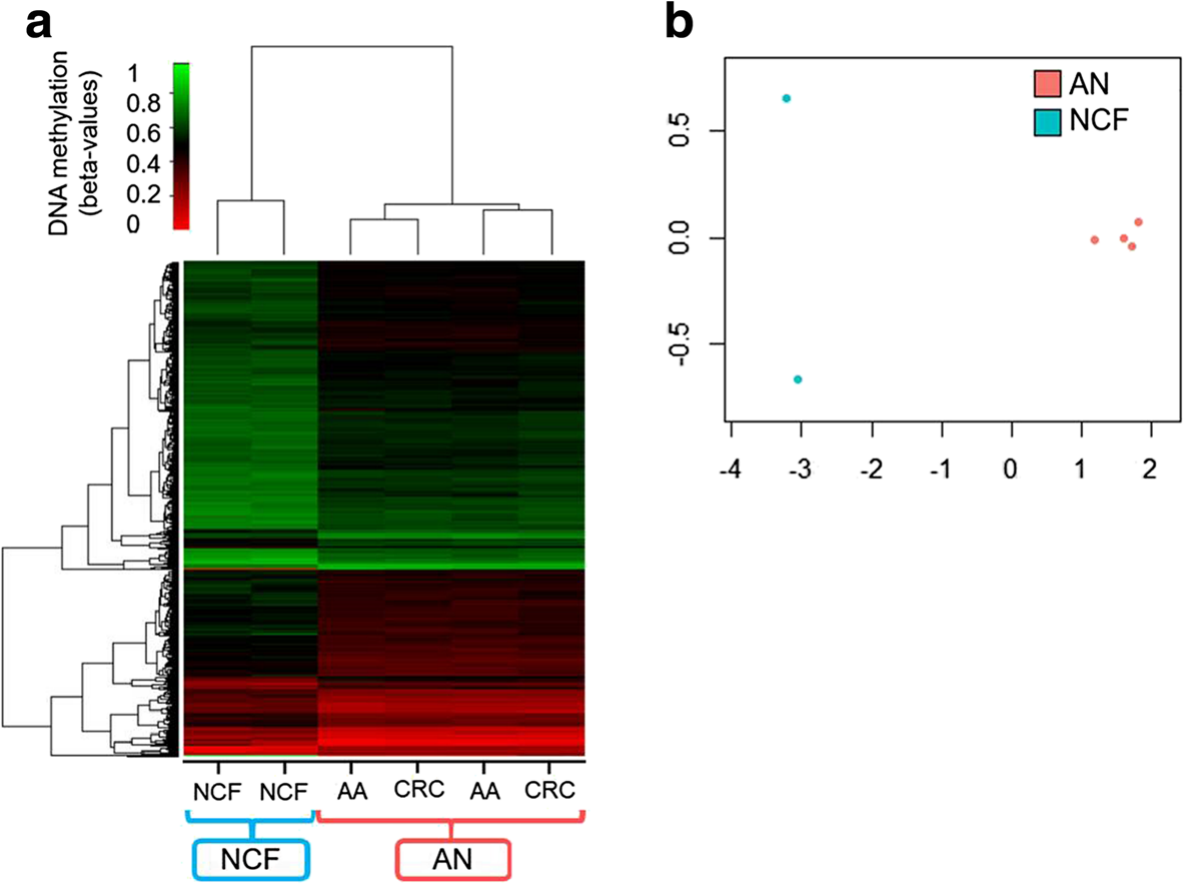
Unsupervised analyses including the 1384 DMPs with |Δbeta| > 0.1. **a** Unsupervised hierarchical clustering and heatmap. Each column represents one pooled sample, and each row represents one of the DMPs (1384). The dendrogram was computed and reordered based on row means. Methylation values are displayed from 0 (red, unmethylated) to 1 (green, fully methylated). **b** Clustering using multidimensional scaling (MDS) based on the 1384 DMPs

Unsupervised clustering performed on DNA methylation values for the top 1384 DMPs identified is presented in Fig. 3a, b. These results highlight the differences between AN and NCF pooled samples and suggest that differential cfDNA methylation profiles obtained with pooled samples can discriminate AN from NCF controls.

**Fig. 4:**
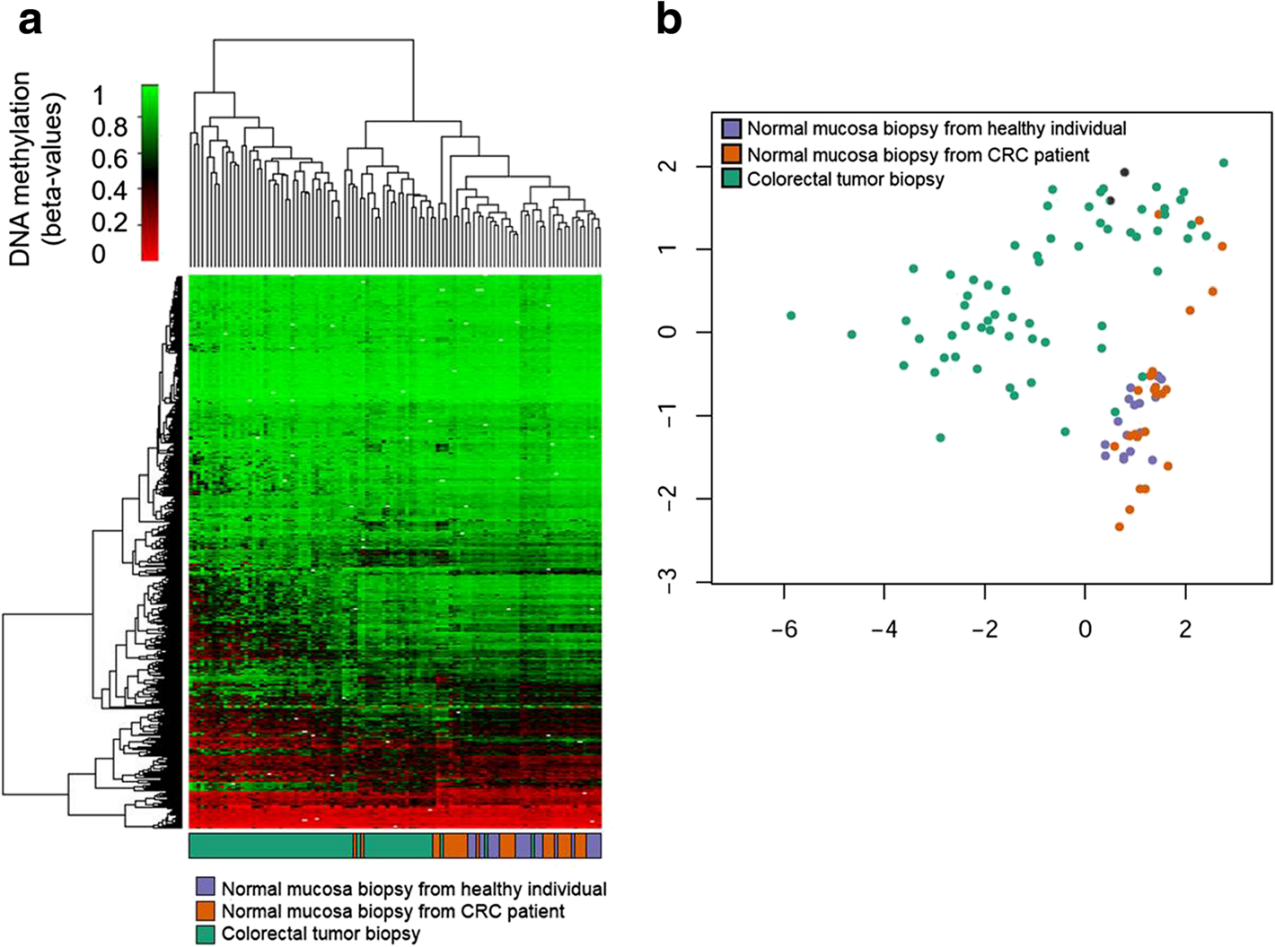
Unsupervised analyses performed on GSE48684 including the 518 DMPs shared by EPIC and 450K arrays. **a** Unsupervised hierarchical clustering and heatmap based on these 518 DMPs. Each column represents one tumor or mucosa sample from GSE48684, and each row represents one CpG. The dendrogram was computed and reordered based on row means. Methylation values are displayed from 0 (red, unmethylated) to 1 (green, fully methylated). **b** Clustering using multidimensional scaling (MDS) on tumor and mucosa samples from GSE48684 based on these 518 DMPs

We further evaluated the DMPs identified in our pooled serum cfDNA samples with dataset GSE48684 consisting of tumor and mucosa tissue samples [14] as a test cohort, restricting the analysis to the 518 DMPs between AN and NCF with |Δbeta| > 0.1 targeted by probes shared by 450K and EPIC arrays. The unsupervised clustering shown in Fig. 4, performed on tumor and mucosa samples from GSE48684 based on our DMPs, reveals that the differential methylation patterns found between AN and NCF cfDNA can also separate tumor tissue from healthy mucosa samples. It is worth to mention that 24 healthy mucosa samples from GSE48684 were normal colon concurrent with CRC and were obtained from the normal-appearing resection margin of the colorectal tumor biopsy [14], as represented in Fig. 4b. This can be related to the fact that a subcluster of mucosa samples partially overlaps with CRC samples. Though this in silico verification is limited, a considerable degree of concordance can be deduced. It should also be mentioned that discrepancy in the frequencies of methylation alterations have been reported in tumor and cfDNA, showing the latter considerably lower frequencies [45]. Hence, array-based strategies that rely on tissue samples for cfDNA methylation marker discovery have the inconvenience of resulting in decreased sensitivity of the selected candidate markers once tested in serum or plasma, limiting their utility as non-invasive tests [46, 47]. An alternative approach for biomarker discovery was accomplished by Heiss et al. that used whole blood. However, these authors indicate that the methylation signature identified in leukocyte DNA may not be specific for CRC, reflecting immune responses [48].

The exploratory nature of this study, with a reduced number of samples, limits further analyses, but offers a new affordable strategy for biomarker discovery, providing an alternative approach to tissue biopsy, reducing costs in microarray-based EWAS. This work should be followed by new studies that include a greater number of pooled serum cfDNA samples and a greater range of colorectal pathologies, allowing a more robust comparison between methylation profiles. Furthermore, differential methylation profiles must be validated in independent serum cfDNA individual samples, using quantitative real-time techniques, with the aim of finding a serum methylation panel for CRC diagnosis and screening.

## Conclusion

As far as we are concerned, this proof-of-principle study is the first to evaluate pooled serum cfDNA profiling on an epigenome-wide scale for CRC biomarker discovery using the MethylationEPIC array. Our data, although preliminary, revealed that the whole epigenome is represented in pooled serum cfDNA samples and that differentially methylated cfDNA profiles can discriminate NCF controls from AN cases (AA or CRC). These results suggest that a pooling strategy using cfDNA may be a valuable source of novel non-invasive methylation biomarkers for CRC early detection and screening. Also, our approach can be translated to the search of biomarkers for other types of tumors, as an affordable alternative approach to tissue biopsy.

## Additional files

Additional file 1:Graphical description of the protocol for DNA sample pooling. ^a^Expected DNA concentration of the pool was calculated as follows: $\frac{\left(\mathrm{limiting}\ \mathrm{ng}\right)\cdotp n}{\left(\mathrm{total}\ \mathrm{volume}\ \mathrm{of}\ \mathrm{the}\ \mathrm{pool}\right)}$ where *n* is the number of individuals included in each pool (10). (PDF 311 kb)

Additional file 2: GenomeStudio software quality control report based on the internal control probes present on the array. (PDF 97 kb)

## Abbreviations

AA: Advanced adenoma
AN: Advanced neoplasia
cfDNA: Circulating cell-free DNA
CRC: Colorectal cancer
DMP: Differentially methylated position
FDR: False discovery rate
NCF: No colorectal findings
