# Supplementary figures and images for: A new approach to epigenome-wide discovery of non-invasive methylation biomarkers for colorectal cancer screening in circulating cell-free DNA using pooled samples
Source: Clin Epigenetics. 2018 Apr 16;10:53. doi: 10.1186/s13148-018-0487-y (PMC5902929; doi:10.1186/s13148-018-0487-y)

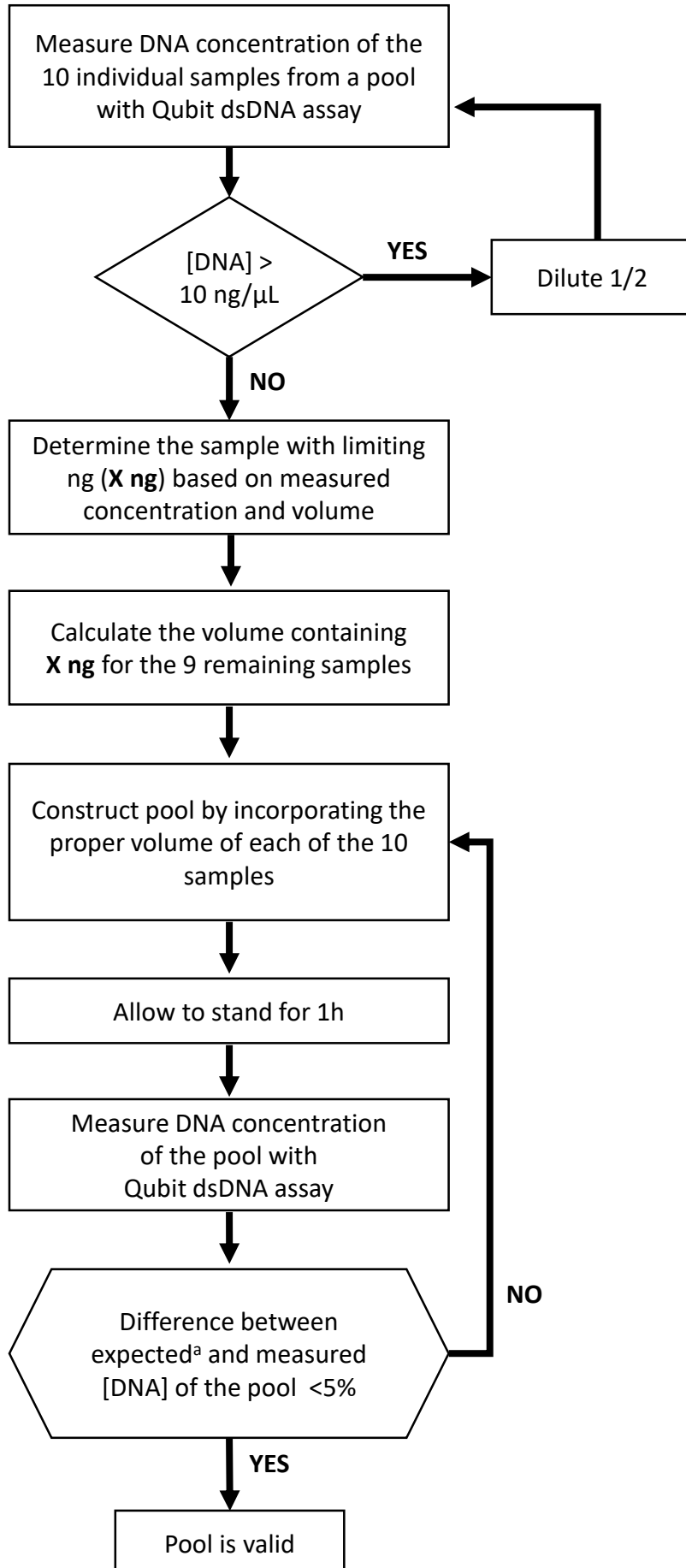

Supplement: Supplementary file 1 — Graphical description of the protocol for DNA sample pooling. aExpected DNA concentration of the pool was calculated as follows: $\frac{\left(\mathrm{limiting}\ \mathrm{ng}\right)\cdotp n}{\left(\mathrm{total}\ \mathrm{volume}\ \mathrm{of}\ \mathrm{the}\ \mathrm{pool}\right)}$limitingng·ntotal volume of the pool where n is the number of individuals included in each pool (10). (PDF 311 kb) [file 13148_2018_487_MOESM1_ESM.pdf]
