# Supplementary material for: A new approach to epigenome-wide discovery of non-invasive methylation biomarkers for colorectal cancer screening in circulating cell-free DNA using pooled samples
Source: Clin Epigenetics. 2018 Apr 16;10:53. doi: 10.1186/s13148-018-0487-y (PMC5902929; doi:10.1186/s13148-018-0487-y)
Supplement: Supplementary file 2 — GenomeStudio software quality control report based on the internal control probes present on the array. (PDF 97 kb) [file 13148_2018_487_MOESM2_ESM.pdf]

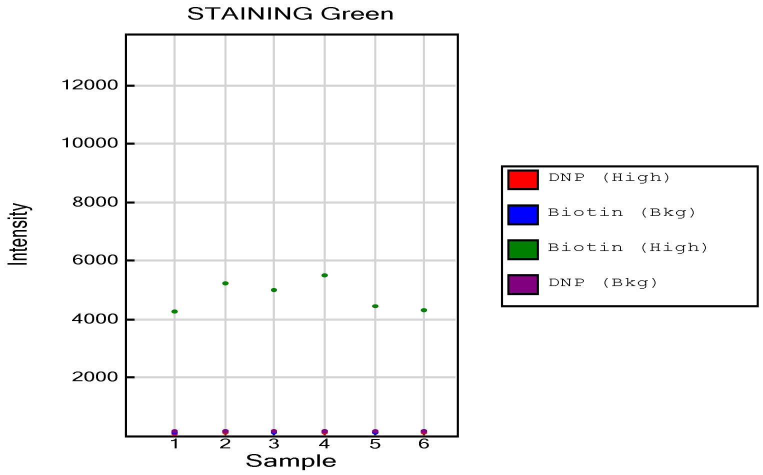
STAINING Green
Intensity
12000
10000
8000
6000
4000
2000
1 2 3 4 5 6
Sample
DNP (High)
Biotin (Bkg)
Biotin (High)
DNP (Bkg)

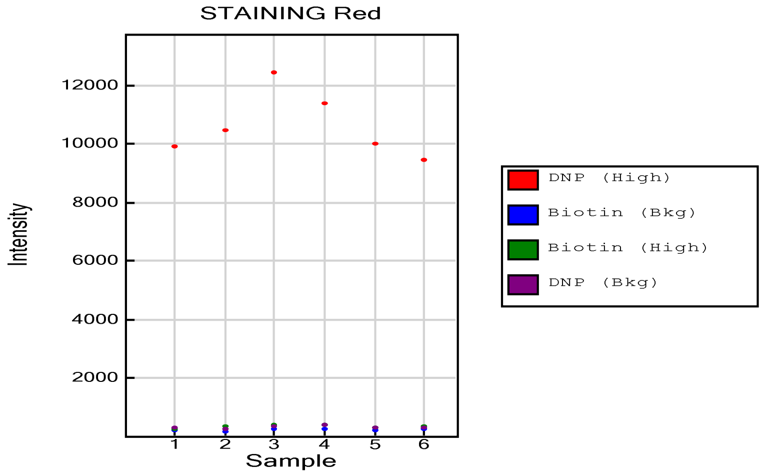
STAINING Red
Intensity
12000
10000
8000
6000
4000
2000
1 2 3 4 5 6
Sample
DNP (High)
Biotin (Bkg)
Biotin (High)
DNP (Bkg)

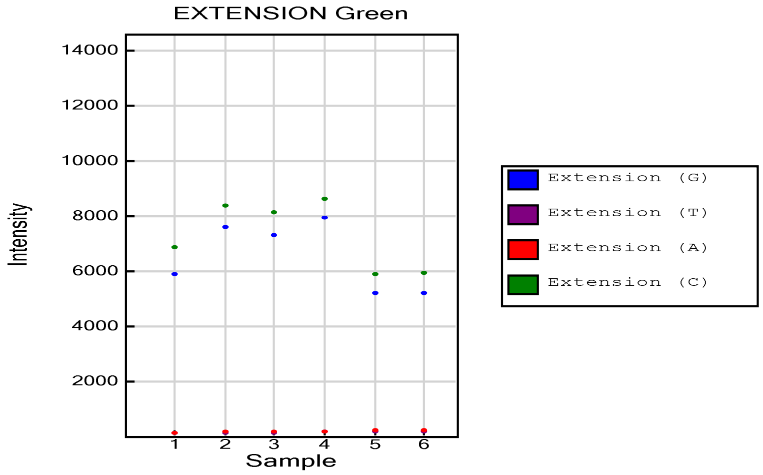
EXTENSION Green
Intensity
14000
12000
10000
8000
6000
4000
2000
1 2 3 4 5 6
Sample
Extension (G)
Extension (T)
Extension (A)
Extension (C)

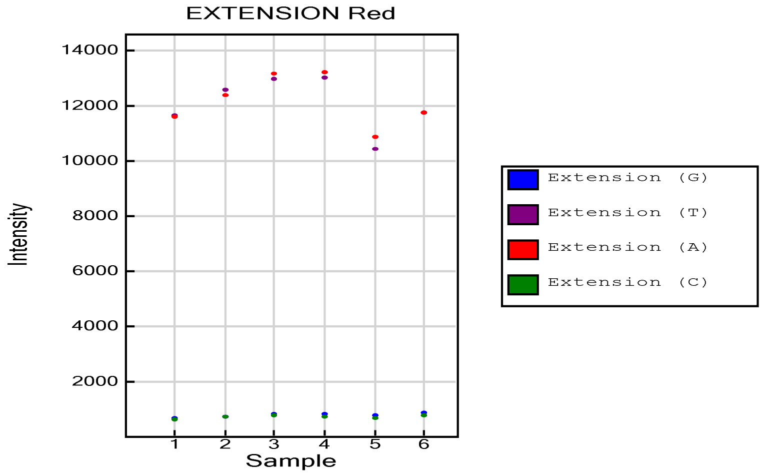
EXTENSION Red
Intensity
14000
12000
10000
8000
6000
4000
2000
1 2 3 4 5 6
Sample
Extension (G)
Extension (T)
Extension (A)
Extension (C)

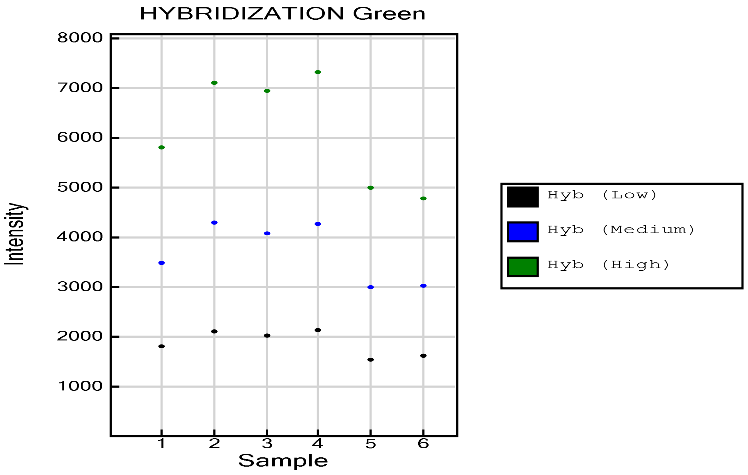
HYBRIDIZATION Green
Intensity
8000
7000
6000
5000
4000
3000
2000
1000
1
2
3
4
5
6
Sample
Hyb (Low)
Hyb (Medium)
Hyb (High)

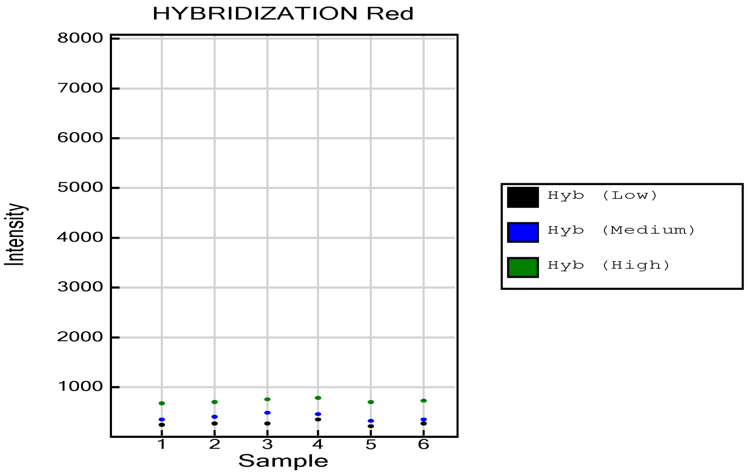
HYBRIDIZATION Red
Intensity
8000
7000
6000
5000
4000
3000
2000
1000
1
2
3
4
5
6
Sample
Hyb (Low)
Hyb (Medium)
Hyb (High)

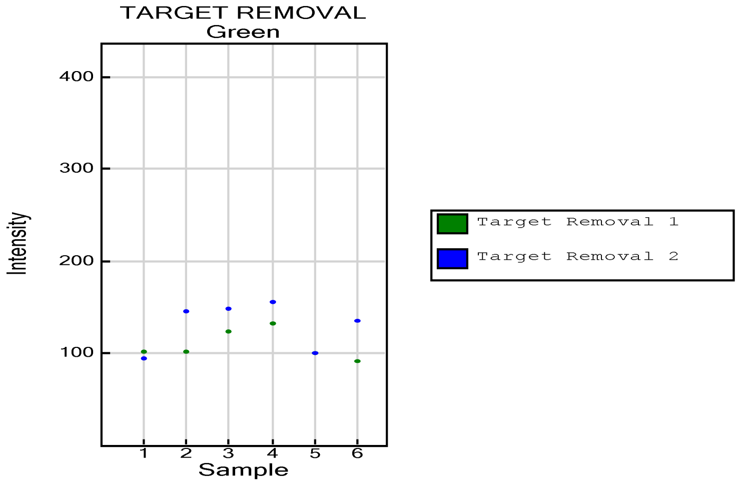
TARGET REMOVAL
Green
Intensity
400
300
200
100
1
2
3
4
5
6
Sample
Target Removal 1
Target Removal 2

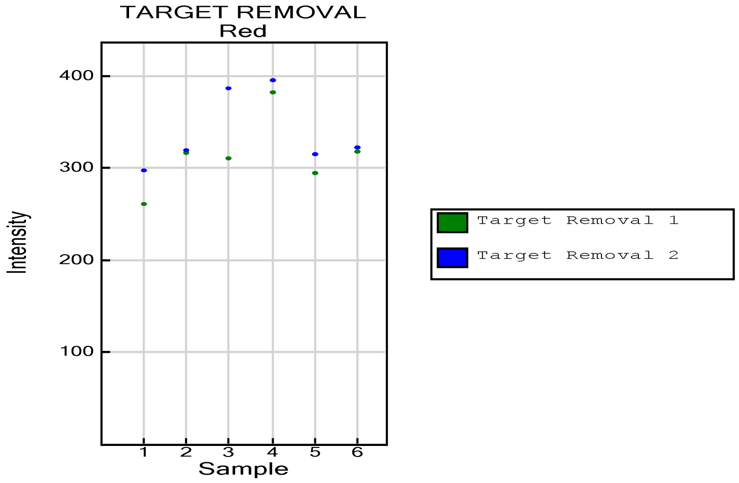
TARGET REMOVAL
Red
Intensity
400
300
200
100
1
2
3
4
5
6
Sample
Target Removal 1
Target Removal 2

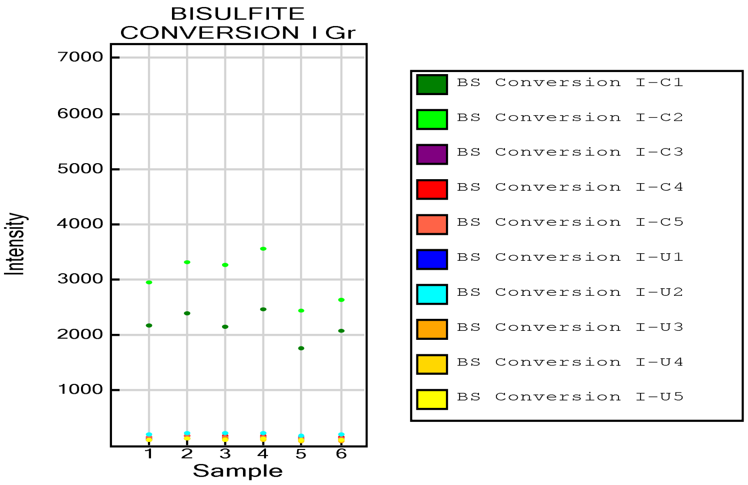
BISULFITE CONVERSION I Gr
Intensity
7000
6000
5000
4000
3000
2000
1000
1 2 3 4 5 6
Sample
BS Conversion I-C1
BS Conversion I-C2
BS Conversion I-C3
BS Conversion I-C4
BS Conversion I-C5
BS Conversion I-U1
BS Conversion I-U2
BS Conversion I-U3
BS Conversion I-U4
BS Conversion I-U5

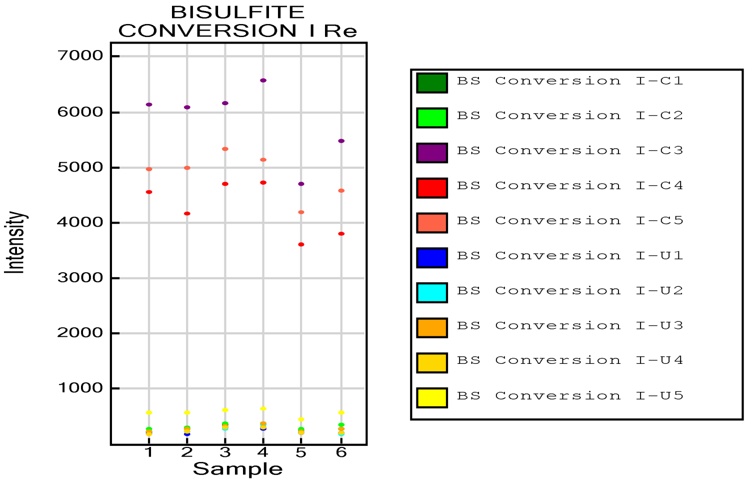
BISULFITE CONVERSION I Re
Intensity
7000
6000
5000
4000
3000
2000
1000
1 2 3 4 5 6
Sample
BS Conversion I-C1
BS Conversion I-C2
BS Conversion I-C3
BS Conversion I-C4
BS Conversion I-C5
BS Conversion I-U1
BS Conversion I-U2
BS Conversion I-U3
BS Conversion I-U4
BS Conversion I-U5

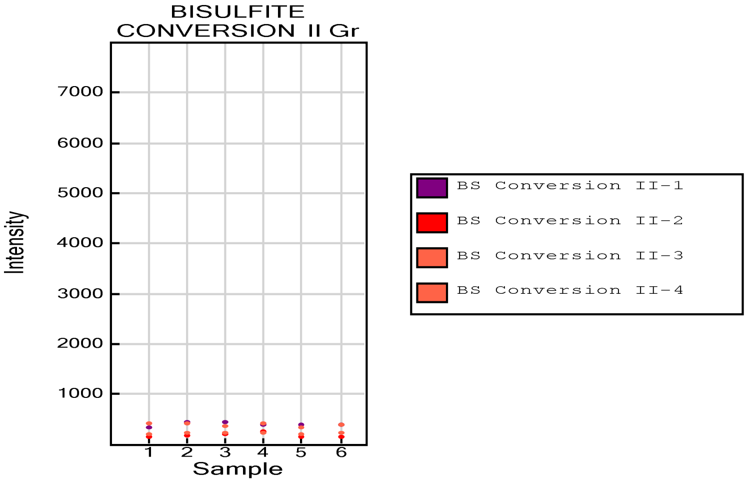
BISULFITE CONVERSION II Gr
Intensity
7000
6000
5000
4000
3000
2000
1000
1 2 3 4 5 6
Sample
BS Conversion II-1
BS Conversion II-2
BS Conversion II-3
BS Conversion II-4

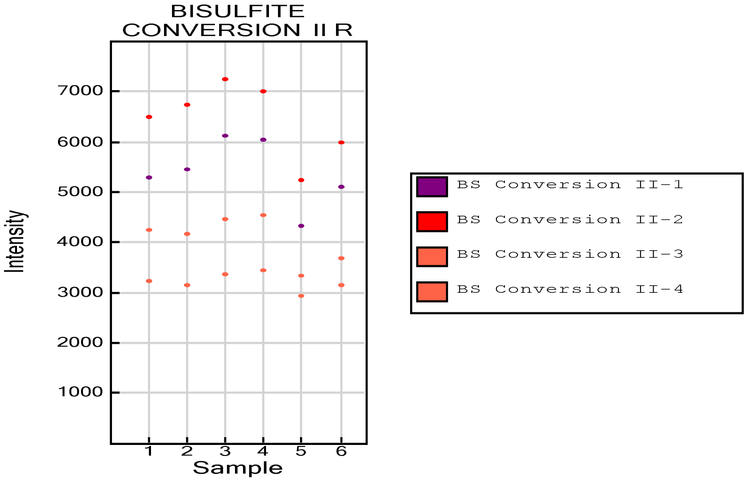
BISULFITE CONVERSION II R
Intensity
7000
6000
5000
4000
3000
2000
1000
1 2 3 4 5 6
Sample
BS Conversion II-1
BS Conversion II-2
BS Conversion II-3
BS Conversion II-4

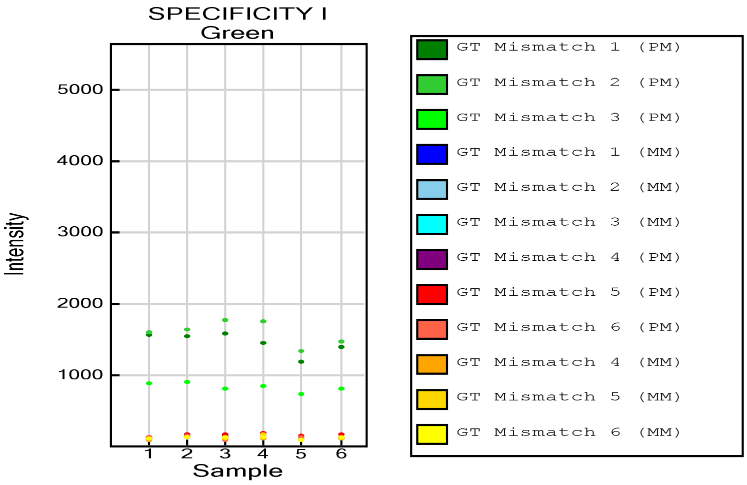
SPECIFICITY I
Green
Intensity
5000
4000
3000
2000
1000
1 2 3 4 5 6
Sample
GT Mismatch 1 (PM)
GT Mismatch 2 (PM)
GT Mismatch 3 (PM)
GT Mismatch 1 (MM)
GT Mismatch 2 (MM)
GT Mismatch 3 (MM)
GT Mismatch 4 (PM)
GT Mismatch 5 (PM)
GT Mismatch 6 (PM)
GT Mismatch 4 (MM)
GT Mismatch 5 (MM)
GT Mismatch 6 (MM)

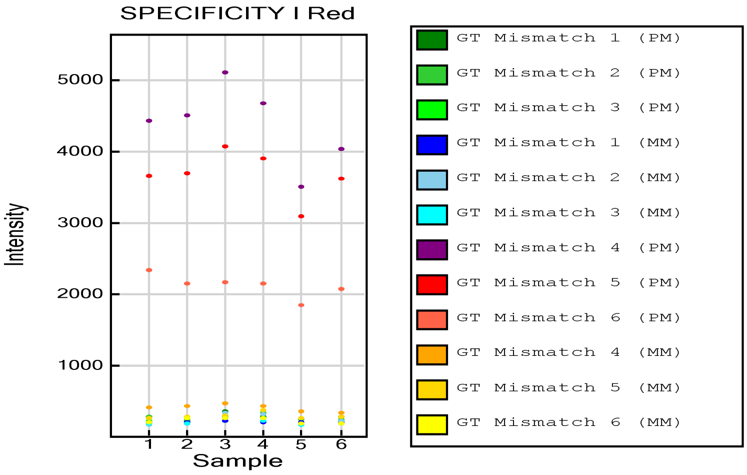
SPECIFICITY I Red
Intensity
5000
4000
3000
2000
1000
1 2 3 4 5 6
Sample
GT Mismatch 1 (PM)
GT Mismatch 2 (PM)
GT Mismatch 3 (PM)
GT Mismatch 1 (MM)
GT Mismatch 2 (MM)
GT Mismatch 3 (MM)
GT Mismatch 4 (PM)
GT Mismatch 5 (PM)
GT Mismatch 6 (PM)
GT Mismatch 4 (MM)
GT Mismatch 5 (MM)
GT Mismatch 6 (MM)

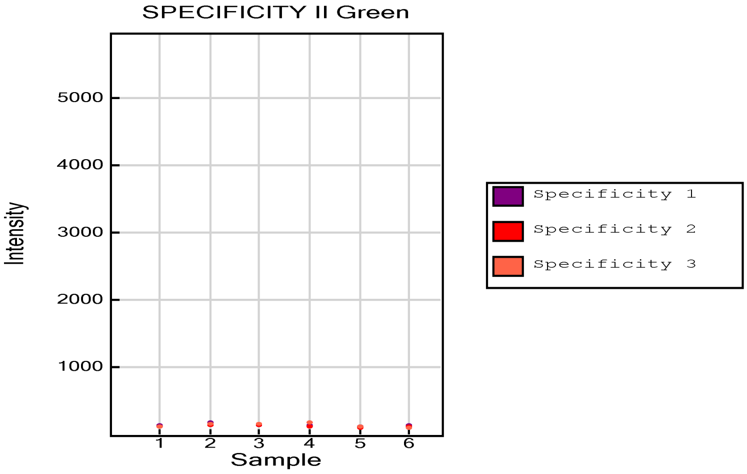
SPECIFICITY II Green
Intensity
5000
4000
3000
2000
1000
1 2 3 4 5 6
Sample
Specificity 1
Specificity 2
Specificity 3

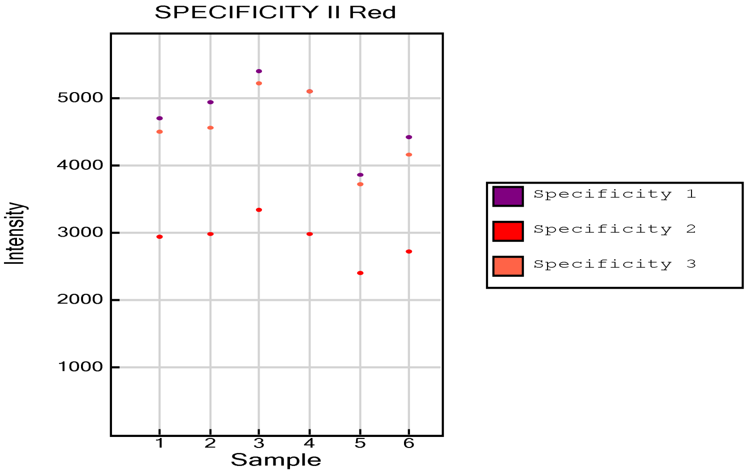
SPECIFICITY II Red
Intensity
5000
4000
3000
2000
1000
1 2 3 4 5 6
Sample
Specificity 1
Specificity 2
Specificity 3

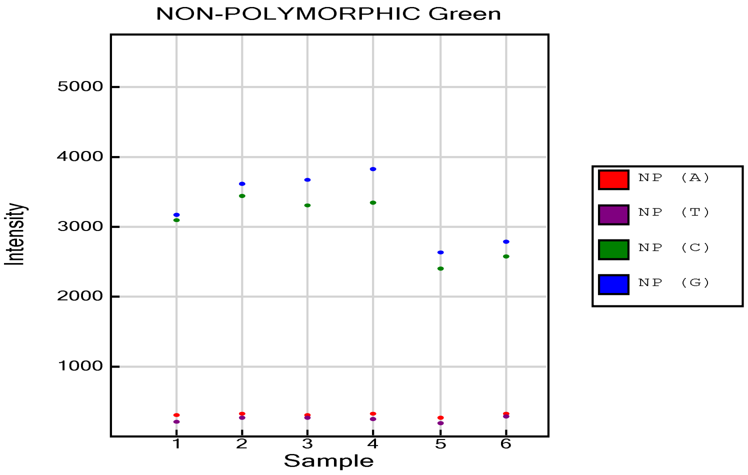
NON-POLYMORPHIC Green
Intensity
Sample
5000
4000
3000
2000
1000
1
2
3
4
5
6
NP (A)
NP (T)
NP (C)
NP (G)

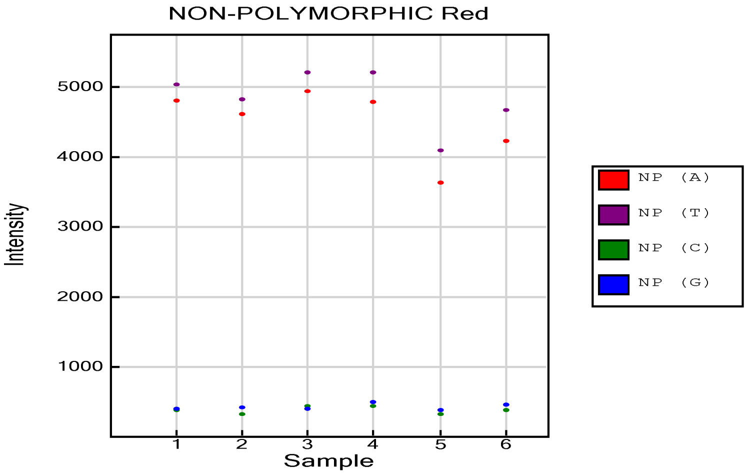
NON-POLYMORPHIC Red
Intensity
Sample
5000
4000
3000
2000
1000
1
2
3
4
5
6
NP (A)
NP (T)
NP (C)
NP (G)

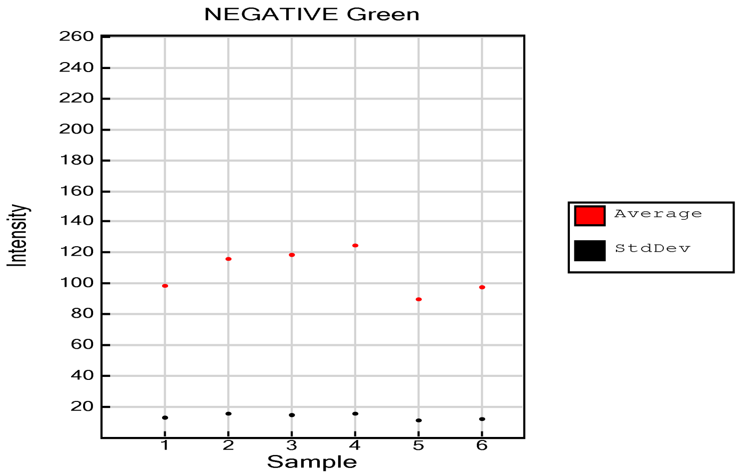
NEGATIVE Green
Intensity
Sample
260
240
220
200
180
160
140
120
100
80
60
40
20
1
2
3
4
5
6
Average
StdDev

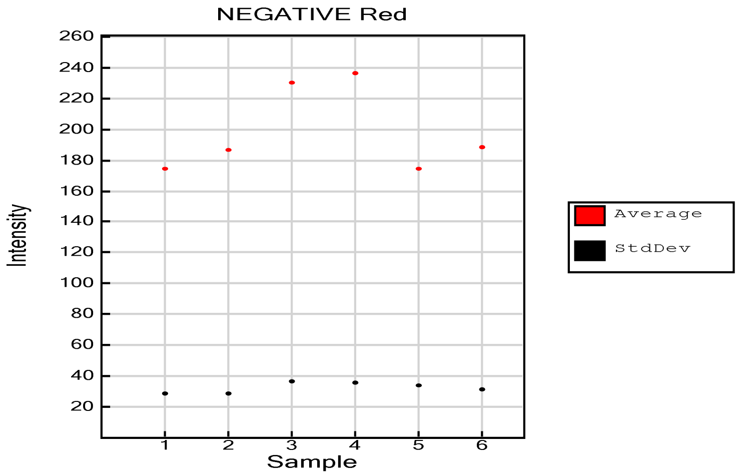
NEGATIVE Red
Intensity
Sample
260
240
220
200
180
160
140
120
100
80
60
40
20
1
2
3
4
5
6
Average
StdDev

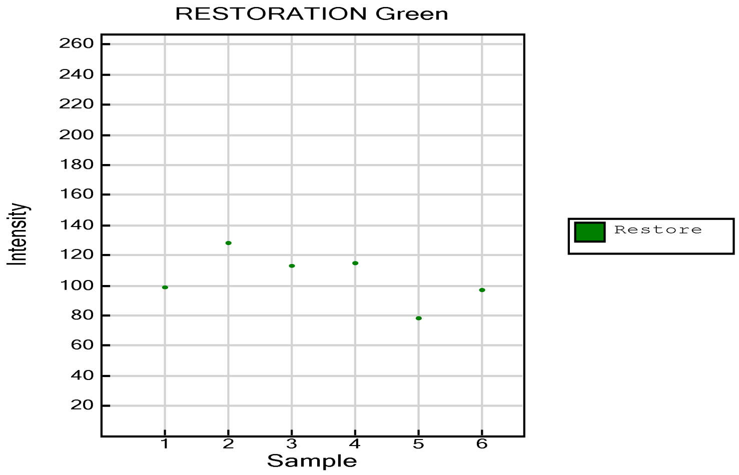
RESTORATION Green
Intensity
Sample
260
240
220
200
180
160
140
120
100
80
60
40
20
1
2
3
4
5
6
Restore

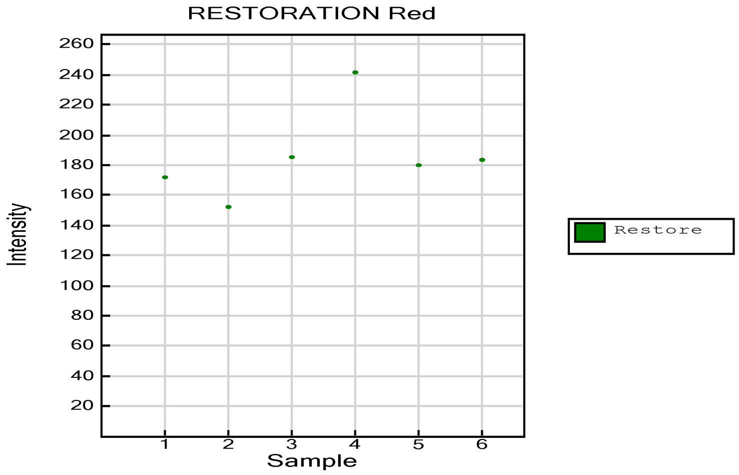
RESTORATION Red
Intensity
Sample
260
240
220
200
180
160
140
120
100
80
60
40
20
1
2
3
4
5
6
Restore
